# Promising Antimicrobial Activities of Essential Oils and Probiotic Strains on Chronic Wound Bacteria

**DOI:** 10.3390/biomedicines13040962

**Published:** 2025-04-14

**Authors:** Mara-Mădălina Mihai, Beatrice Bălăceanu-Gurău, Alina Maria Holban, Cornelia-Ioana Ilie, Romina Maria Sima, Cristian-Dorin Gurău, Lia-Mara Dițu

**Affiliations:** 1 Department of Oncologic Dermatology, “Elias” Emergency University Hospital, “Carol Davila” University of Medicine and Pharmacy, 020021 Bucharest, Romania; mara.mihai@umfcd.ro; 2 Research Institute of the University of Bucharest, Department of Botany-Microbiology, Faculty of Biology, University of Bucharest, 050663 Bucharest, Romania; alina_m_h@yahoo.com (A.M.H.); lia_mara_d@yahoo.com (L.-M.D.); 3 Department of Science and Engineering of Oxide Materials and Nanomaterials, Faculty of Applied Chemistry and Materials Science, National University of Science and Technology POLITEHNICA Bucharest, 011061 Bucharest, Romania; cornelia_ioana.ilie@upb.ro; 4 National Centre for Micro and Nanomaterials and National Centre for Food Safety, National University of Science and Technology POLITEHNICA Bucharest, 060042 Bucharest, Romania; 5 “Bucur Maternity” Obstetrics and Gynecology Discipline, “Carol Davila” University of Medicine and Pharmacy, 020021 Bucharest, Romania; romina.sima@umfcd.ro; 6 Orthopedics and Traumatology Clinic, Clinical Emergency Hospital, “Carol Davila” University of Medicine and Pharmacy, 020021 Bucharest, Romania; cristian-dorin.gurau@drd.umfcd.ro

**Keywords:** chronic wounds, skin microbiota, biofilm, essential oils, probiotics, antimicrobial resistance, *Staphylococcus aureus*, *Pseudomonas aeruginosa*

## Abstract

**Background:** Disruption of the natural balance of the skin microbiota can impair wound healing and contribute to chronic infections. Identifying the bacterial species involved and understanding their antimicrobial susceptibility profiles are essential for guiding treatment, especially given the growing threat of antibiotic resistance. **Methods:** This study characterized the virulence and antibiotic resistance phenotypes of 43 bacterial strains isolated from chronic wounds. The antimicrobial activity of selected essential oils (sandalwood, ylang-ylang, sage, cajeput, and juniper), pharmaceutical products (propolis tinctures, usnic acid), and probiotic lactic acid bacteria strains (*Lactobacillus* spp., *Lactococcus lactis*) was assessed using qualitative and quantitative assays, including MIC, MICBA, and co-culture evaluations. **Results:** Gram-positive strains were more sensitive to essential oils than Gram-negative strains, with sandalwood, ylang-ylang, and propolis tincture showing the strongest antibacterial effects. These agents also showed significant biofilm inhibition. Probiotic strains exhibited antimicrobial activity against *Staphylococcus aureus* and *Morganella morganii*, with *Lactobacillus paracasei* and *Lactobacillus rhamnosus* being particularly effective in reducing bacterial growth and adhesion in vitro. **Conclusions:** Essential oils and probiotic strains demonstrate promising antimicrobial effects against chronic wound pathogens and may serve as alternative or adjunctive treatments to antibiotics. Further clinical research and standardization are necessary to establish their safety, efficacy, and optimal application protocols.

## 1. Introduction

The skin microenvironment comprises various cellular types—such as keratinocytes, fibroblasts, and immune cells—that play critical roles in preserving skin integrity and defending against pathogenic insults [1,2]. Among the primary defense mechanisms are antimicrobial peptides (AMPs) and lipid molecules, including sphingosine and dihydrosphingosine, which are derived from precursors such as sphingomyelin, glucosylceramides, and phospholipids. These biomolecules possess selective antimicrobial activity and contribute to the elimination of specific bacterial species [1,2,3].

Alongside these endogenous defenses, commensal microorganisms—such as bacteria (*Staphylococcus epidermidis*, *Cutibacterium acnes*) and fungi (*Malassezia* spp.)—provide colonization resistance by outcompeting or inhibiting pathogenic species, including *Staphylococcus aureus* and *Pseudomonas aeruginosa* [3].

Disruptions in this microbial equilibrium, termed dysbiosis, have been implicated in the pathogenesis of several skin disorders. Moreover, the capacity of pathogens to organize into biofilms—structured microbial communities encased in a self-produced matrix—contributes to the chronicity and persistence of skin infections [4,5]. Biofilms have been identified in more than 90% of chronic wounds, whereas they are detected in only 6% of acute wounds [6]. Chronic wound biofilms often include not only *S. aureus* and *P. aeruginosa*, but also *Escherichia coli*, *Klebsiella pneumoniae*, *Peptoniphilus*, *Enterobacter*, *Stenotrophomonas*, *Finegoldia*, and *Serratia* species [5,7]. Beyond the presence of pathogens, the cumulative bacterial load can further impair wound healing [5,7].

In recent years, the growing prevalence of multidrug-resistant bacteria has posed a significant global health challenge. This trend is largely attributed to the overuse and misuse of antibiotics, leading to diminished therapeutic efficacy of many conventional antimicrobials [8,9,10]. Consequently, the management of infections caused by resistant pathogens is increasingly difficult, resulting in elevated rates of morbidity, mortality, and a reduced quality of life for affected patients [10]. Additionally, these infections have a considerable economic impact due to prolonged hospitalizations and the increased cost of care [10].

**Figure 1:**
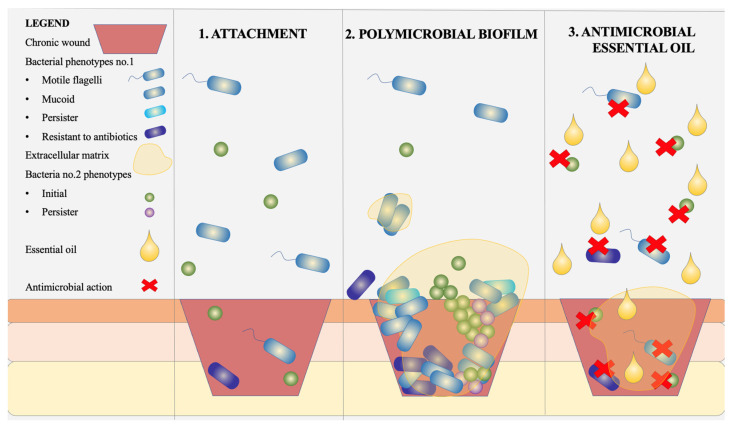
Schematic representation of the stages involved in chronic wound colonization and the effect of essential oils. (**1**) Attachment: Motile and non-motile bacterial cells (both Gram-positive and Gram-negative phenotypes) initially adhere to the wound surface; (**2**) polymicrobial biofilm: Bacterial proliferation and biofilm maturation occur with the development of an extracellular matrix, comprising mixed phenotypes including antibiotic-resistant, mucoid, and persister cells; (**3**) antimicrobial essential oil exposure: Essential oils penetrate the biofilm, exerting bactericidal activity on both planktonic and biofilm-embedded cells, resulting in visible zones of antimicrobial action.

In response to these challenges, alternative antimicrobial solutions are being actively pursued. Among them, essential oils and aromatic plant extracts have gained prominence due to their rich content of antimicrobial phytochemicals [10,11,12]. Ideally, these agents could act across various bacterial phenotypes, including those involved in biofilm formation (Figure 1). Furthermore, there has been a notable surge in the use of probiotics and prebiotics as adjunctive therapies due to their favorable safety profile and ability to modulate microbiota [13]. Although their mechanisms are not yet fully elucidated, these agents are believed to restore microbial balance and influence immune responses by interacting with epithelial cells and modulating both innate and adaptive immunity in a manner similar to commensal bacteria [13,14].

Our study aimed to establish the phenotypic profiles of virulence and antibiotic resistance of bacterial strains isolated from chronic wounds and to investigate the antimicrobial activity of essential oils and other pharmaceutical products, as well as of probiotic lactic acid bacterial strains.

## 2. Materials and Methods

Agar, Nutrient Broth, Sabouraud Glucose Agar, Luria–Bertani (LB) broth, Man–Rogosa–Sharpe (MRS) broth, usnic acid, dimethyl sulfoxide (DMSO), ethanol, methanol, acetic acid, and crystal violet were purchased from Sigma-Aldrich (Darmstadt, Germany). Tween 80, sage (*Salvia officinalis*) essential oil, sandalwood (*Santalum album*) essential oil, ylang-ylang (*Cananga odorata*) essential oil, juniper berry (*Juniperus communis*) essential oil, and cajeput (*Melaleuca leucadendron*) essential oil were acquired from Roth (Karlsruhe, Germany). All chemicals were used without further purification. All strains tested in this study were obtained from the Microorganisms Collection of the Microbiology Department, Faculty of Biology, University of Bucharest.

### 2.1. Characterization of Pathogenic Strains

A total of 44 bacterial strains isolated from chronic wounds were analyzed, including *S. aureus* (*n* = 32), *Enterococcus faecium* (*n* = 2), *Enterococcus faecalis* (*n* = 1), *Morganella morganii* (*n* = 1), *Serratia marcescens* (*n* = 4), and *P. aeruginosa* (*n* = 3).

#### 2.1.1. Virulence Factor Production

Strains were assessed for production of DNase, caseinase, gelatinase, lipase, esculinase, lecithinase, amylase, and hemolysins using agar-based assays (Table 1). Results were determined by visual inspection of color changes, halo formation, and hemolysis patterns.

#### 2.1.2. Semiquantitative Assessment of Biofilm Formation on Inert Substratum

Biofilm development was assessed over 24, 48, and 72 h using 96-well plates cultivation in broth media and the crystal violet microtiter method for evaluation of the biofilm developed on inert substratum. Absorbance was measured at 490 nm, using a BIOTEK SYNERGY-HTX ELISA multi-mode reader (Agilent Technologies, Winooski, VT, USA) [15,16].

### 2.2. Antimicrobial Activity of Essential Oils and Products

Essential oils and pharmaceutical agents (Table 2) were tested for antimicrobial activity using Spot diffusion for qualitative assessment and serial two-fold microdilution for determining minimum inhibitory concentration (MIC) and minimum biofilm eradication concentration (MBEC). Absorbance was measured at 620 nm for MIC and 490 nm for MBEC [15].

### 2.3. Probiotic Potential of Lactic Acid Bacteria (LAB) Strains

LAB strains (Table 3) were cultured and fractionated into free cells supernatants (SN) and cell suspensions (CS) after centrifugation (10 min, 6000 rpm) (Table 4). The CS fraction was washed two times in PBS and recentrifuged in order to prepare the cell suspensions with a final density of 1.5 × 10^8^ CFU/mL (corresponding to McFarland standard density). The SN fractions were used in two forms: SNs with Ph = 7–7.2 (adjusted with sterile NaOH 10% solution) and SNs with acid pH (corresponding to LAB fresh cultures).

Bacteriocin activity of SNs (both free pH and neutral pH) was assessed via spot-on-lawn assays, using the adapted disk-diffusion method.

The modulation of pathogenic strain growth and multiplication was assessed using a co-cultivation assay in 24-well plates, followed by incubation for 24 h at 37 °C, plating, and colony-forming unit per milliliter (CFU/mL) determination. The co-cultivation scheme was evaluated in two different broth media: MRS (de Man, Rogosa, and Sharpe medium, a selective culture medium for LAB, Sigma-Aldrich, Darmstadt, Germany) and LB (Luria–Bertani, a widely used medium for bacterial growth, Thermo Fisher, Waltham, MA, USA). For each pathogenic strain, the bacterial suspensions in PBS with a standardized density of 1.5 × 10^8^ CFU/mL were prepared. The co-cultivation schemes are represented in Table 5, with each co-cultivation variant being performed in duplicate.

### 2.4. Bacterial Adherence to Cell Substrate

HEp-2 cells were used to evaluate bacterial adherence in the presence of essential oils, probiotic SN, or combinations. The adherence index and pattern were determined post-staining. The HEp-2 cells were cultured in Dulbecco’s Modified Eagle’s Medium (Gibco, Waltham, MA, USA) supplemented with 10% FBS (Gibco, Waltham, MA, USA), 100 U/mL penicillin, and 100 μg/mL streptomycin, using 6-well plates and a humidified atmosphere (with 5% CO_2_) at 37 °C. After the cells reached the 80% confluence, the cell substrate was inoculated with *S. aureus 19*, *S. aureus 22, P. aeruginosa 1, P. aeruginosa 3,* and *M. morganii* bacterial suspensions, in different combinations with EO5, EO6, and *L. rhamnosus MF9* SN fraction (SN5) (Table 6).

### 2.5. Statistical Analysis

All experiments were conducted in triplicates, comprising three independent biological replicates and three technical replicates for each experimental condition. Data are presented as mean values ± standard deviation (SD), where applicable. Standard deviations have now been included in the graphs where relevant. In addition, statistical significance was assessed using one/two-way ANOVA followed by Tukey’s or Dunnett’s post hoc test, with *p*-values < 0.05 considered statistically significant. Statistical analyses were performed using GraphPad Prism 10.4.1 from GraphPad Software (San Diego, CA, USA). These statistical assays ensure improved reproducibility, transparency, and reliability of the results presented.

## 3. Results

We focused on identifying the factors that influence the colonization of specific pathogens, as well as the microbiome imbalances that facilitate pathogen colonization and biofilm formation, which can delay healing. In exploring alternative approaches for treating skin infections, we examined the use of essential oils and certain probiotics for localized application.

### 3.1. Characterization of the Phenotypic Profile of Virulence of Pathogenic Strains

The pathogenic strains, isolated from chronic wounds, were obtained from the strain collection of the Faculty of Biology, Department of Botany and Microbiology, in Bucharest, Romania. A total of 43 pathogenic strains, both Gram-positive and Gram-negative, have been characterized: 32 strains of *S. aureus* (S.a.1–S.a.32), 2 strains of *E. faecium* (E.c.1, E.c.2), 1 strain of *E. faecalis* (E.c.3), 1 strain of *M. morganii* (M.m.), 4 strains of *S. marscescens* (S.m.1–S.m.4), and 3 strains of *P. aeruginosa* (P.a.1–P.a.3).

#### 3.1.1. Phenotypic Determination of the Production of Soluble Virulence Factors

**Figure 2:**
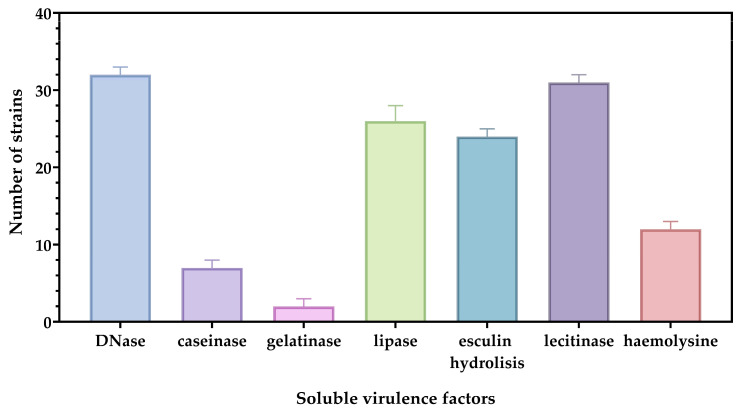
Graphical representation of the pattern of soluble virulence factors highlighted in the main species involved in skin pathologies. The differences between soluble virulence factors are statistically quantified using one-way ANOVA and Tukey’s multiple comparisons tests. The results are considered statistically significant (*p* < 0.0001).

The enzymes associated with soluble virulence factors promote the tissue integrity destruction, creating favorable conditions for infection progression but also bacterial survival in the external environment, thereby increasing the risk of transmission to new susceptible hosts. In our study, 70% of pathogenic strains were found to produce DNase and lecithinase (Figure 2). Lecithin (phosphatidylcholine) is a key component of cell membranes. The enzymatic activity of lecithinases leads to membrane disruption, cell lysis, and tissue damage, contributing to bacterial virulence [17].

In addition, 55% of pathogenic strains showed lipase and esculinase production (Figure 2). In terms of hemolysis, most strains exhibited γ hemolysis. While it is not a major virulence factor, esculin hydrolysis may aid in survival in diverse environments; instead, the lipases induce tissue damage and facilitate infections, but also contribute to bacterial adhesion and biofilm development [18,19].

#### 3.1.2. Phenotypic Assessment of Bacterial Adherence to Inert Substrate

**Figure 3:**
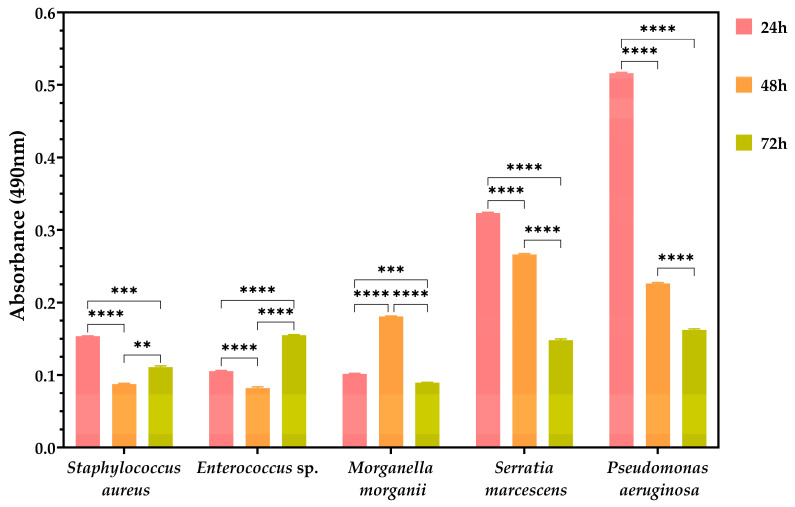
Adhesion to inert substrate. The ability of different bacterial species to adhere to inert surfaces was assessed by measuring absorbance at 490 nm at 24 h, 48 h, and 72 h. The differences between groups are statistically quantified using two-way ANOVA and Tukey’s multiple comparisons tests. The data results are considered statistically significant (** *p* < 0.018; *** *p* ≤ 0.004; **** *p* < 0.0001).

In general, most bacterial species exhibited a high adhesion capacity to the inert substrate within the first 24 h, followed by a decline in absorbance values (Figure 3). This decrease indicates a reduced ability to form mature biofilms. However, some exceptions were observed, such as *M. morganii* at 24 h and *Enterococcus* spp. at 72 h, which exhibited the highest levels of adhesion and were also capable of forming biofilms (Figure 3). The adhesion to the inert substrate primarily relies on adhesins, which facilitates the development of biofilms.

### 3.2. Assessment of the Antimicrobial Activity of Commercial Essential Oils and Pharmaceutical Products

#### 3.2.1. Qualitative Assessment of Bacterial Inhibition

**Figure 4:**
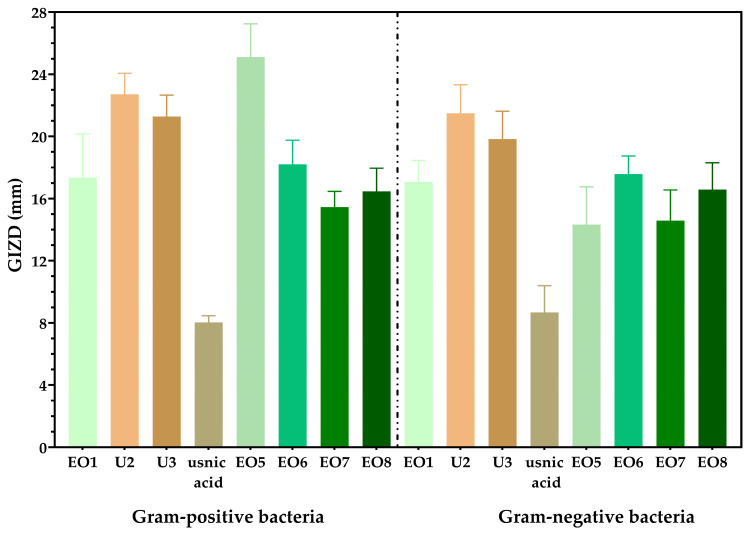
Graphical representation of the arithmetic mean values of the growth diameters of the inhibition zones (GIZDs) for the tested species. The results are expressed in millimeters (mm). The differences between samples are statistically analysed using one-way ANOVA and Tukey’s multiple comparisons tests. The data are considered statistically significant (*p* < 0.001).

We observed that Gram-positive strains exhibit larger inhibition zone diameters compared to Gram-negative bacteria (Figure 4). While the antibacterial activity of propolis and sage oil is already well-established, our study demonstrates the effectiveness of sandalwood, ylang-ylang, juniper, and cajeput oils (Figure 4). In terms of sensitivity to Gram-positive pathogenic strains, sandalwood essential oil and 10% propolis tincture were found to be the most effective. For Gram-negative pathogenic strains, the most effective options were 10% propolis tincture and 30% propolis skin spray (Figure 4).

**Figure 5:**
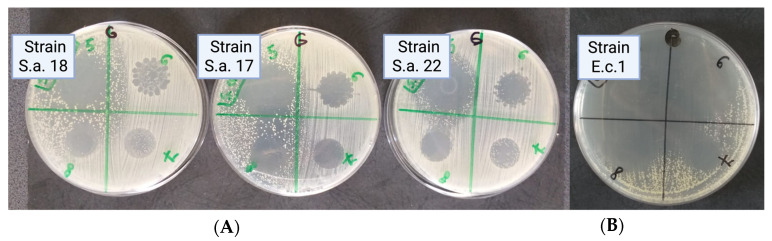
(**A**) *S. aureus* culture with synergic inhibition zone between sandalwood and cajeput diluted essential oils; (**B**) *Enterococcus* spp. culture with synergic inhibition zone between sandalwood, ylang-ylang, juniper berry, and cajeput diluted essential oils.

We observed a synergistic effect between sandalwood oil (cod 5 in Figure 5) and cajeput oil (cod 8 in Figure 5). In the case of *Enterococcus* sp., a synergistic effect of sandalwood, ylang-ylang, juniper, and cajeput oils were displayed.

#### 3.2.2. Quantitative Assessment of Bacterial Inhibition

Regarding quantitative analysis, sandalwood oil exhibited the highest effectiveness with low MIC values, expressed as a percentage. For Gram-negative strains, sandalwood oil showed the lowest MIC values, ranging from 0.0078% to 1%. Propolis tincture, propolis skin spray, and sandalwood essential oil also confirmed the qualitative results for Gram-positive strains, displaying MIC values between 0.25% and 0.125%.

#### 3.2.3. Quantitative Assessment of Biofilm Eradication in Vitro

**Figure 6:**
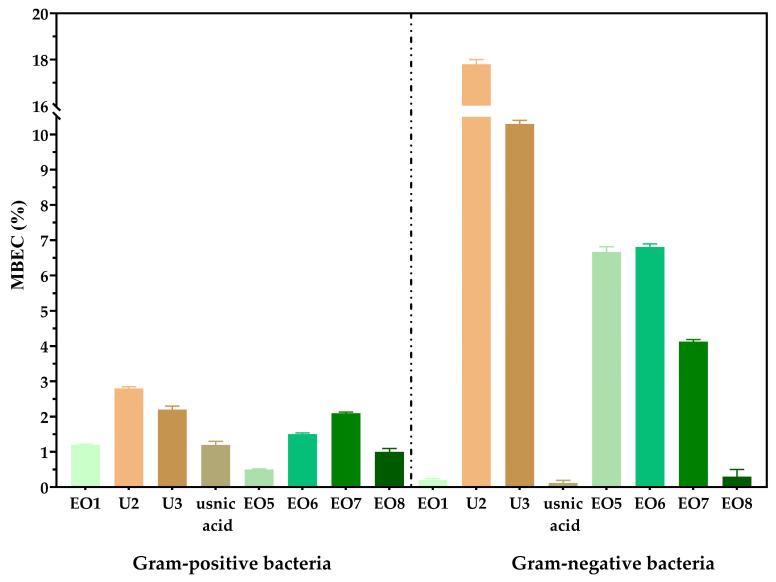
Graphical representation of the minimum biofilm eradication concentration—MBEC (%)—of the tested strains. The differences between samples are statistically analyzed using one-way ANOVA and Tukey’s multiple comparisons tests. The data are considered statistically significant (*p* < 0.001).

The analysis of the minimum biofilm eradication concentration (MBEC %) revealed distinct patterns for Gram-negative and Gram-positive bacteria (Figure 6). The graphical representation shows that, on average, Gram-negative bacteria exhibit higher MBEC values compared to Gram-positive bacteria (Figure 6). While essential oils were effective in disrupting biofilms formed by Gram-positive bacteria, probiotic supernatants showed limited biofilm eradication activity, particularly for Gram-negative strains. The efficiency of the tested plant products in eradicating biofilms was greater for Gram-positive bacteria, with lower concentrations required to disrupt biofilm formation compared to Gram-negative bacteria. Cajeput and sage essential oils were effective in eliminating biofilms of Gram-negative bacteria (Figure 6).

### 3.3. The Evaluation of Probiotic Potential of Selected Lactic Acid Bacteria Strains

The LAB cultures used in this study included *L. acidophilus* ATCC 4356, *L. plantarum* 8, *L. paracasei* MC1C, *L. lactis* DP1, and *L. rhamnosus* MF9. The SN fractions were tested for antagonistic activity against indicator microorganisms using the spot-on-lawn method [20].

The impact of normal pH and pH-adjusted probiotics on the tested strains is depicted in Table 7 No significant inhibitory effect was observed for the supernatant fractions, as evidenced by the absence of a measurable zone of inhibition diameters (Table 7).

For instance, in the case of *S. marcescens* 1, the supernatant SN1 (derivate from *L. acidophilus* 4356 culture) exhibited a mild inhibitory effect at normal pH. However, when the pH was adjusted, no zone of inhibition was observed. These findings suggest that the inhibitory effect against *S. marcescens* is primarily attributed to the lactic acid produced by the probiotic at normal pH (pH 4.5).

On the other hand, for *S. aureus* 6, SN3 (derivate from *L. paracasei* MC1C culture) demonstrated a slight inhibitory effect at both normal pH and adjusted pH (pH 7). This suggests that the inhibitory effect is driven by factors such as lactic acid produced under normal pH conditions, as well as other inhibitory substances like bacteriocins or microbial peptides. Some strains displayed slight inhibitory effects on agar medium, but overall, the growth inhibition was not significant.

The impact of probiotics on the tested strains generally shows limited inhibitory effects on both Gram-positive and Gram-negative bacteria, with some exceptions observed, particularly against Gram-positive strains, particularly *S. aureus*.

**Figure 7:**
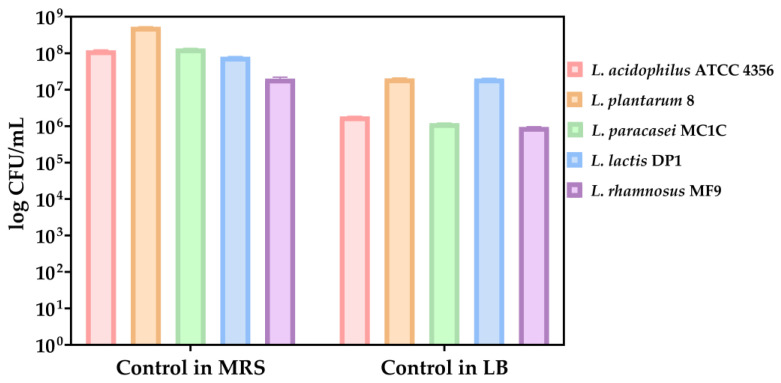
Bacterial growth (CFU/mL, logarithmic scale) of probiotic strains after 24 h incubation in MRS and LB media. Initial suspensions were standardized to 0.5 McFarland (approx. 1.5 × 10^8^ CFU/mL); increases reflect post-incubation growth in nutrient-rich conditions. Values represent means ± standard deviation from three biological replicates. Axis uses logarithmic scaling for clarity. The data results are considered statistically significant (*p* < 0.0001).

As growth controls, the LABs have been cultivated in both MRS and LM medium. A better growth was observed in MRS medium after the analysis of growth control of lactic acid bacteria strains cultivated in both MRS and LB medium (Figure 7).

**Figure 8:**
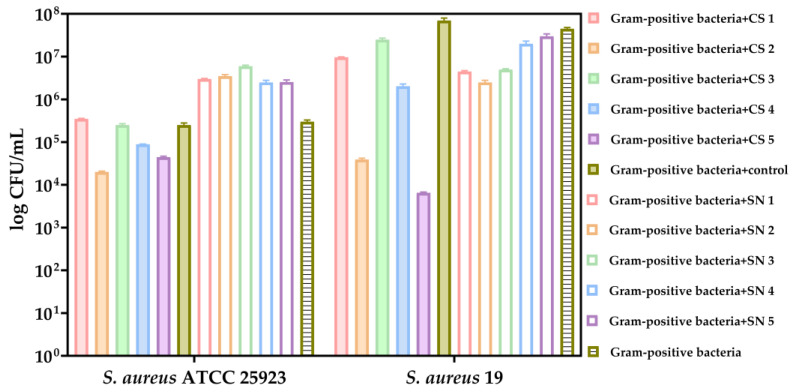
Bacterial growth (CFU/mL, logarithmic scale) of the selected Gram-positive bacteria (*S. aureus* ATCC 25923 and *S. aureus* 19) after 24 h co-cultivation in MRS medium with LAB fractions. Co-cultures included either LAB cell suspensions (CS1–CS5) or supernatants (SN1–SN4). Controls included *S. aureus* monoculture and unexposed LAB. The data results are considered statistically significant (*p* < 0.0001).

In comparison to the growth control of Gram-positive pathogenic species, it can be observed that the cell suspensions of LAB exerted inhibitory effects on the growth of the tested *Staphylococcus* species in the favorable environment provided by MRS medium (Figure 8). Notably, a pronounced inhibitory effect was evident in SC5 (*L. rhamnosus*), which was isolated from newborn feces (Figure 8).

**Figure 9:**
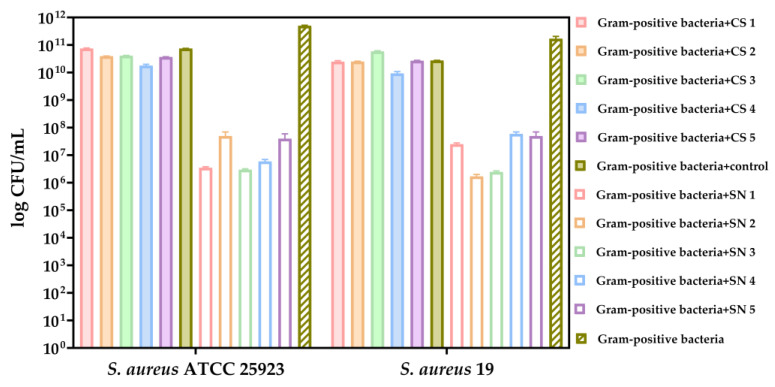
Bacterial growth (CFU/mL, logarithmic scale) of the selected Gram-positive bacteria (*S. aureus* ATCC 25923 and *S. aureus* 19) after 24 h co-cultivation in LB medium with LAB cell suspensions (CS) and supernatants (SN). Monoculture controls and untreated controls are included for comparison. The data results are considered statistically significant (*p* < 0.0001).

Our results showed that the supernatants of lactic acid bacteria exhibited inhibition of both tested Gram-positive strains, in contrast to the cell suspensions (Figure 9). Particularly noteworthy is SN3 (*L. paracasei*), which was isolated from newborn feces and showed a significant inhibitory effect (Figure 9).

The growth of the Gram-negative bacteria tested was not quantifiable in the MRS medium. The cell suspensions of lactic acid bacteria did not show any inhibitory effect on the Gram-negative strains.

**Figure 10:**
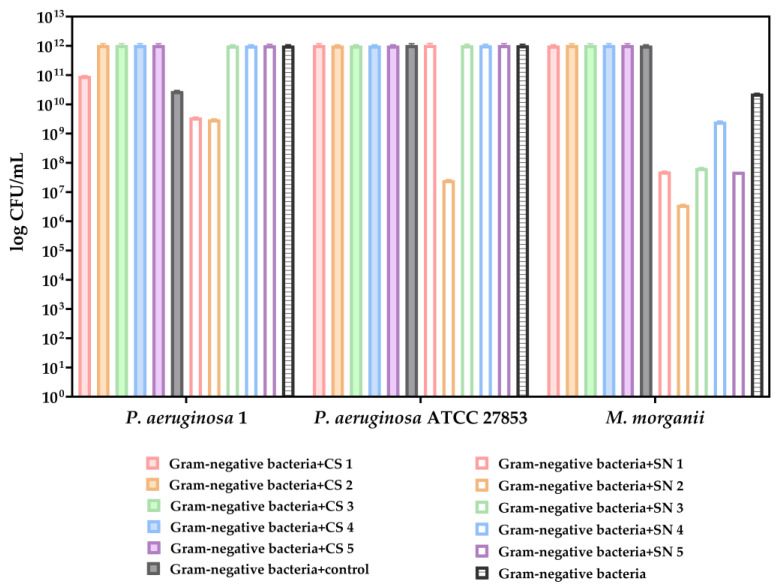
Graphical representation of CFU/mL (logarithmic scale) resulting from co-cultivations in LB medium of lactic acid bacteria supernatants and cell suspensions with tested Gram-negative bacteria. The data results are considered statistically significant (*p* < 0.0001).

Our results indicated that the supernatants of lactic acid bacteria exhibited inhibitory activity only against *M. morganii* (Figure 10). Among the lactic acid bacteria strains tested, SN2 (*L. plantarum* 8) isolated from fermented vegetable products showed the highest effectiveness in inhibiting the growth of *M. morganii* (Figure 10).

### 3.4. Cell Substrate Adherence Assay of Pathogenic Bacteria in the Presence of Essential Oils Alone or in Combination with L. Rhamnosus SN Fraction

**Figure 11:**
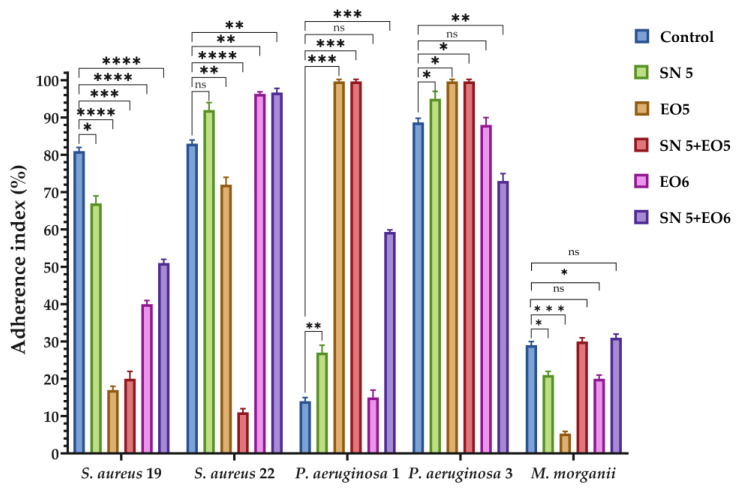
Graphical representation of the cell substrate adhesion index of the tested strains. The differences between samples are statistically analyzed using two-way ANOVA and Dunnett’s multiple comparisons tests. The data results are considered statistically significant (ns—not significant; * *p* < 0.049, ** *p* ≤ 0.067, *** *p* ≤ 0.005, **** *p* < 0.0001).

It can be noted that EO5 (sandalwood essential oil) exhibits greater effectiveness in terms of cell substrate adhesion index for both Gram-positive bacteria (*S. aureus* 19) and Gram-negative bacteria (*M. morganii*) (Figure 11). In the case of Gram-positive bacteria, the combination of sandalwood essential oil (EO5) at sub-inhibitory concentration with *L. rhamnosus* (SN5) supernatant fraction showed inhibitory effects on both strains (Figure 11). However, when essential oils were used in combination with *L. rhamnosus* supernatant, they demonstrated a relatively weak effect on *P. aeruginosa* (Figure 11). Moreover, the use of sandalwood essential oil (EO5) alone proved to be highly effective against *M. morganii* (Figure 11).

## 4. Discussions

Essential oils are volatile compounds naturally synthesized by plants, recognized for their characteristic aromas and flavors [12]. These substances are produced in specialized cellular structures such as glandular trichomes, secretory cavities, and resin ducts [12]. Present as liquid droplets, they are distributed across various plant organs, including leaves, stems, flowers, fruits, bark, and roots [12]. Although dominated by two or three primary constituents that make up 20–70% of the oil, they are chemically complex mixtures comprising mainly terpenes, terpenoids, and phenylpropanoids [12]. Additional components like fatty acids, oxides, and sulfur derivatives further enrich their chemical diversity [12].

Traditional methods for extracting essential oils include hydro-distillation, steam distillation, dry distillation, and cold pressing, while modern laboratory techniques such as microwave-assisted extraction and supercritical fluid extraction are also employed [12]. Fermentation, mechanical pressing, solvent extraction, and enzymatic hydrolysis are additional approaches to isolate essential oils [12].

The antimicrobial activity of essential oils is primarily attributed to their bioactive compounds, particularly terpenoids and phenolic derivatives, which disrupt microbial cell membranes, impair enzymatic function, and interfere with cell signaling pathways, thereby inhibiting growth or inducing cell death [21]. These compounds exhibit activity against a broad spectrum of microorganisms, including both Gram-positive and Gram-negative bacteria, which highlights their potential as alternative antimicrobial agents [21].

Given these properties, essential oils are widely applied in therapeutic and medical settings [12]. Gram-positive bacteria are generally more susceptible to essential oils due to their cell wall structure, particularly the presence of lipoteichoic acids that facilitate oil penetration [22]. Bioactive components of essential oils can bind to the bacterial surface and insert into the phospholipid bilayer, leading to structural disruption, metabolic interference, and cell death [23]. This compromise in membrane integrity results in leakage of essential intracellular components such as proteins, sugars, ATP, and DNA [23]. Moreover, essential oils inhibit ATP production and enzyme systems, which can lead to electrolyte imbalance and microbial lysis [23]. At minimum inhibitory concentrations (MICs), significant damage to the bacterial membrane has been observed [23].

Usnic acid, a lichen-derived secondary metabolite, has shown antimicrobial activity specifically against Gram-positive species such as *S. aureus, E. faecalis,* and *E. faecium*, but it is ineffective against Gram-negative bacteria and fungi [24].

*Cananga odorata* (ylang-ylang) essential oil contains multiple bioactive compounds, including linalool, citral, borneol, camphor, and linalyl acetate [25]. These constituents exert antimicrobial effects by damaging membrane structure, reducing ATP levels, altering membrane potential, and decreasing cytoplasmic pH [22]. This oil is commonly obtained by water or steam distillation of the plant’s flowers [25]. Ylang-ylang oil is used in dermatology to manage acne, seborrheic imbalance, dermatitis, eczema, insect bites, and for general skin care [26]. It is also used in aromatherapy, purported to relieve depression, respiratory issues, hypertension, and anxiety [27]. Notable allergenic compounds include alpha-farnesene, germacrene, beta-caryophyllene, benzyl acetate, benzyl benzoate, and linalool [27].

A study investigating the antimicrobial potential of *C. odorata* leaf extracts revealed that methanolic extracts exhibited stronger antibacterial activity than petroleum ether or chloroform extracts, with Gram-positive bacteria being more susceptible than Gram-negative strains [25]. Common assessment methods included the disc diffusion test and MIC determination [25]. Notably, *S. aureus* was highly sensitive, with MIC90 values of 0.23 mg/mL against both reference and clinical isolates [28], while *E. coli* and *P. aeruginosa* were resistant, showing no inhibition even at 27.12 mg/mL [28].

Cajeput oil, known for its antiseptic properties since the 18th century, contains active compounds like 1,8-cineole, linalool, and terpinen-4-ol [29]. It is used to treat dermatological conditions such as acne, psoriasis, insect bites, and blemishes with antimicrobial effects similar to tea tree oil [26,29]. At concentrations of 0.2–0.4%, cajeput oil inhibits several Gram-positive species, and at concentrations of 0.4–0.6%, it also acts against Gram-negative bacteria [29].

Sage essential oil, obtained from *Salvia officinalis* or *Salvia grandiflora*, is rich in camphor, thujone, and 1,8-cineole—components responsible for its antibacterial properties [30]. It demonstrates activity against both Gram-positive and Gram-negative bacteria, including *E. coli, Bacillus subtilis, Salmonella* spp., *S. aureus*, *S. epidermidis*, *Streptococcus mutans*, and *Shigella sonnei* [30].

Propolis owes its antimicrobial capacity to the presence of flavonoids, aromatic acids, and esters, with compounds like galangin, chrysin, pinocembrin, and pinostrobin showing particularly strong activity [31]. It is more effective against Gram-positive bacteria due to its phenolic-rich composition [31].

Sandalwood essential oil from *Santalum album* is composed primarily of sesquiterpenoids like α-santalol and β-santalol, which give it anti-inflammatory and antiseptic properties used in treating acne and psoriasis [32,33]. It is widely used for conditions such as eczema, wounds, fungal infections, and inflammation [26]. This oil demonstrates robust antibacterial effects, even against resistant strains such as MRSA and VRSA, along with moderate activity against select Gram-negative species [32].

The *Juniperus* genus includes slow-growing shrubs and trees known for their aromatic wood and essential oil content, found across diverse climates [34]. Juniper essential oil, derived from leaves, wood, or berries, is used in dermatology for acne, dermatitis, eczema, psoriasis, and wounds [26,35]. It also contains non-volatile agents like podophyllotoxin, useful in psoriasis therapy [35].

The antimicrobial activity of *Juniperus excelsa* and *Juniperus sabina* essential oils has been tested against several pathogens, including *E. coli*, *H. influenzae*, *S. sonnei, Y. enterocolitica, S. pneumoniae,* and *S. aureus*, with notable effects against *E. coli* and *Y. enterocolitica* [36]. *J. sabina* oil was especially effective against *H. influenzae, Bacillus subtilis,* and *S. aureus*, while *J. excelsa* showed efficacy against *Clostridium perfringens* and moderate action against other bacterial and fungal strains [34].

Probiotics are live microorganisms that provide health benefits when consumed in sufficient quantities [37]. In skin health, they help restore microbial balance, enhance immunity, and competitively inhibit pathogenic colonization [37]. Probiotics also produce bioactive compounds that interfere with pathogen growth and quorum sensing [37].

In wound care, probiotics—particularly strains of *Lactobacillus* and their fermented products—have been explored to modulate skin microbiota and promote healing [38]. Commonly used strains include *L. rhamnosus, L. acidophilus*, *L. plantarum*, *L. casei*, *L. delbrueckii, B. infantis, B. animalis subsp. lactis*, and *B. longum*, along with species like *Propionibacterium acidilactici, Lactococcus lactis, Leuconostoc mesenteroides, Bacillus subtilis*, and yeasts like *Saccharomyces* spp. [37].

Growing scientific evidence supports probiotic use in treating various conditions, including antibiotic-associated diarrhea, *C. difficile* infection, irritable bowel syndrome, inflammatory bowel disease, and wound healing [39]. Probiotic cosmetics have emerged as a novel class aiming to support skin homeostasis by promoting beneficial microbes and inhibiting pathogens [40].

Al-Ghazzewi and Tester demonstrated that konjac glucomannan hydrolysate, in combination with strains like *L. acidophilus, L. casei, L. plantarum, L. gasseri*, and *L. lactis*, inhibits *C. acnes* growth in vitro [41]. Bateni et al. reported that a topical formulation containing konjac glucomannan hydrolysate improved acne severity after 20–40 days of application [42]. Lopes et al. showed that multiple probiotics, including *L. acidophilus La-5, L. delbrueckii, L. paracasei La-26,* and *B. animalis B-12*, adhered to human keratinocytes and significantly reduced *C. acnes* biofilm formation [43].

In a clinical trial by Blanchet-Réthoré et al., a lotion containing heat-inactivated *Lactobacillus johnsonii* NCC 533 reduced *S. aureus* colonization in patients with mild to moderate atopic dermatitis over a 3-week treatment period, suggesting its utility in microbiome-targeted skincare [44].

## 5. Conclusions

The growing resistance of pathogens to conventional antibiotics underscores the urgent need for alternative antimicrobial strategies, particularly in chronic wound management. Our study demonstrates that sandalwood essential oil, ylang-ylang essential oil, and propolis tincture exhibit notable antibacterial effects, especially against Gram-positive strains. Furthermore, probiotic strains—specifically the supernatant of *L. paracasei* MC1C (SN3) and the cell suspension of *L. rhamnosus* MF9 (SC5)—significantly inhibited the growth and adhesion of Gram-positive pathogens. While a synergistic effect between essential oils and probiotic supernatants could not be conclusively confirmed, combinations involving sandalwood oil and SN fractions showed enhanced inhibitory effects. These findings support the potential of essential oils and probiotics as promising therapeutic options for chronic skin infections, though further studies are necessary to standardize formulations, dosing, and application methods for clinical use.

## Figures and Tables

**Table 1:** Overview of media and incubation conditions.

Application	Medium	Incubation Conditions
Pathogenic strains (initial culture)	Nutrient agar	24 h, 37 °C
Hemolysis test	Blood agar	24–48 h, 37 °C
esculin hydrolysis	esculin and ferric citrate medium	24 h, 37 °C
Lecithinase Production	agar supplemented with egg yolk (2.5%)	24 h, 37 °C
Lipase Production	agar supplemented with 1% Tween 80	24 h, 37 °C
Protease Production (caseinase, gelatinase)	agar supplemented with gelatin or milk casein	24 h, 37 °C
Amylase Production	agar supplemented with 1% starch	24 h, 37 °C
Biofilm formation on inert substratum	Nutrient broth (96-well plates)	24/48/72 h, 37 °C
Co-cultivation assay	LB/MRS broth	24 h, 37 °C
Bacterial adherence to HEp-2 cells	Dulbecco’s Modified Eagle’s Medium + 10% fetal serum bovine (FBS)	2 h, 37 °C, 5% CO_2_

**Table 2:** Essential oils and products tested.

Product	Type	Concentration	Solvent
Sage essential oil (EO1)	Essential oil	25% (1/4 diluted)	Ethanol
Sandalwood essential oil (EO5)	Essential oil	25% (1/4 diluted)	Ethanol
Ylang-ylang essential oil (EO6)	Essential oil	25% (1/4 diluted)	Ethanol
Juniper berry essential oil (EO7)	Essential oil	25% (1/4 diluted)	Ethanol
Cajeput essential oil (EO8)	Essential oil	25% (1/4 diluted)	Ethanol
Propolis tincture (U2)	Pharmaceutical	10%	Ethanol
Propolis spray (U3)	Pharmaceutical	30%	Not specified
Usnic acid	Lichen-derived	5 mM	DMSO

**Table 3:** LAB strains and sources.

No.	Lactic Acid Bacteria Strains	Source
1	*Lactobacillus acidophilus* ATCC 4356	Commercial strain, included in the Microbial collection of the Microbiology Department, Faculty of Biology, University of Bucharest
2	*Lactobacillus plantarum* 8	Isolated from fermented vegetables and included in the Microbial collection of the Microbiology Department, Faculty of Biology, University of Bucharest
3	*Lactobacillus paracasei* MC1C	Isolated from newborn feces and included in the Microbial collection of the Microbiology Department, Faculty of Biology, University of Bucharest
4	*Lactococcus lactis* DP1	Isolated from dental plaque and included in the Microbial collection of the Microbiology Department, Faculty of Biology, University of Bucharest
5	*Lactobacillus rhamnosus* MF9	Isolated from newborn feces and included in the Microbial collection of the Microbiology Department, Faculty of Biology, University of Bucharest

**Table 4:** The LAB strains and the fractions codification.

No.	LAB Strain	Supernatant (SN)	Cell Suspension (CS)
1	*L. acidophilus* ATCC 4356	SN1	CS 1
2	*L. plantarum* 8	SN2	CS 2
3	*L. paracasei* MC1C	SN3	CS 3
4	*L. lactis* DP1	SN4	CS 4
5	*L. rhamnosus* MF9	SN5	CS 5

**Table 5:** The co-cultivation schemes for pathogenic strains growth inhibition in the presence of LAB fractions.

1.6 mL broth medium + 200 µL SN1 + 200 µL pathogenic strain suspension	1.6 mL broth medium + 200 µL SN2 + 200 µL pathogenic strain suspension	1.6 mL broth medium + 200 µL SN3 + 200 µL pathogenic strain suspension	1.6 mL broth medium + 200 µL SN4 + 200 µL pathogenic strain suspension	1.6 mL broth medium + 200 µL SN5 + 200 µL pathogenic strain suspension	1.8 mL broth medium + 200 µL pathogenic strain suspension (growth control)
1.6 mL broth medium + 200 µL CS1 + 200 µL pathogenic strain suspension	1.6 mL broth medium + 200 µL CS 2 + 200 µL pathogenic strain suspension	1.6 mL broth medium + 200 µL CS 3 + 200 µL pathogenic strain suspension	1.6 mL broth medium + 200 µL CS4 + 200 µL pathogenic strain suspension	1.6 mL broth medium + 200 µL CS5 + 200 µL pathogenic strain suspension	1.8 mL broth medium + 200 µL pathogenic strain suspension (growth control)

**Table 6:** Different combinations used in the adherence experiment.

Samples	Experimental Combinations
Adherence control	200 μL: bacterial suspension in phosphate buffer saline (PBS) (1.5 × 10^8^ CFU/mL)
Combination 1	200 μL bacterial suspension in PBS (1.5 × 10^8^ CFU/mL) + 100 μL free cells SN5 (pH 7)
Combination 2	200 μL bacterial suspension in PBS (1.5 × 10^8^ CFU/mL) + 20 μL EO5 or EO6 (dilution 1/5 in PBS)
Combination 3	200 μL bacterial suspension in PBS (1.5 × 10^8^ CFU/mL) + 100 μL free cells SN5 (pH 7) + 20 μL EO5 or EO6 (dilution 1/5 in PBS)

**Table 7:** The effect of pH-normal and pH-adjusted probiotics on the tested strains.

Tested Strains	SN1 pH	SN1 pH aj	SN2 pH	SN2 pH aj	SN3 pH	SN3 pH aj	SN4 pH	SN4 pH aj	SN5 pH	SN5 pH aj
*S. aureus* ATCC 25923	–	–	–	–	–	–	–	–	–	–
*S. aureus* 6	–	–	±	±	±	±	±	±	–	–
*S. aureus* 11	–	–	–	–	–	–	–	–	–	–
*S. aureus* 15	–	–	–	–	–	–	–	–	–	–
*S. aureus* 21	–	–	–	–	–	–	–	–	–	–
*P. aeruginosa* 1	±	±	±	±	±	–	–	–	–	–
*S. marscescens* 1	±	–	±	–	±	–	–	–	–	–
*E. faecalis* 3	±	–	±	–	±	–	±	–	±	–
*M. morganii*	±	±	±	±	±	±	±	±	–	–

“–” indicates no inhibitory effect; “±” indicates partial or variable inhibitory effect.

## Data Availability

This review summarizes data reported in the literature and it does not report primary data.
